# A Preliminary Study on “Personalised Treatment” against *Varroa destructor* Infestations in Honey Bee (*Apis mellifera*) Colonies

**DOI:** 10.3390/ani13060987

**Published:** 2023-03-08

**Authors:** Rajmund Sokół, Maria Michalczyk

**Affiliations:** Department of Parasitology and Invasive Diseases, Faculty of Veterinary Medicine, University of Warmia and Mazury in Olsztyn, Oczapowskiego 13 Street, 10-718 Olsztyn, Poland

**Keywords:** personalised medicine, *Varroa destructor*, amitraz, queen isolation, brood

## Abstract

**Simple Summary:**

The *Varroa destructor* mite is a severe problem for the development of beekeeping in many parts of the world. The presented study concerns the control of this harmful nuisance mite via a disease management protocol for the first time. Poor field conditions made it possible to evaluate the protocol’s effectiveness against mites in a natural and uncontrolled way. “Personalised” (tailored) applications consist of adjusting the number of control agents with different doses of amitraz to the number of detected parasite females in a given colony, taking into account a specific brood of queen bees (sisters) with a specific brood area. We showed that the number of treatments did not affect egg laying (brood surface) by mother sisters. We confirmed that amitraz should be increased by more than the number of mites found. The best results were obtained by repeating the procedure four times. We confirmed that effectiveness depends on the degree of Varroa infestation in a given family and the treatment of mites before the procedure. This procedure enables the protocol (personalisation) to effectively control of the impact of parasites on the bee colony. In such a procedure, one of the reasons for efficacy is the genetic conditions related to the reproductive potential of queens resulting from the bee breed. Based on new research, the presented study may change the overall effects of Varroa treatment in bee colonies.

**Abstract:**

The requirement for the protection of bee colonies against *Varroa destructor* invasions has been noted by many breeders and is included as an aspect of the development of beekeeping. This research aimed to check the effect of the development of a colony exposed to laying eggs (brood surface) by queen bees with similar chemical potential (sisters) on the effect of a preparation combating *V. destructor* depending on the number of mites found in a given colony. We chose this as a standard model of conduct that treats each bee colony as one organism subjected to individual parasite control. For this purpose, we created a bee colony with a mother-of-one breeding line and fertilised drones from one colony. Infection with *V. destructor* occurred naturally and uncontrollably. Without interfering with the colony’s development, the frame insulator helped each colony’s brood (mothers’ reproductive potential) and the initial and final individuals from the mites themselves. The study was carried out in four species (two control species and two species with up to 20 and over 21 mites, respectively). Treatments with amitraz to combat damage were divided into four treatment subgroups: two treatments every four days or four treatments every two days. We observed the number of individuals that were protected in all subgroups in the average brood area. The reproductive potential of the sisters’ mothers did not change after the treatments with amitraz, which indicated that amitraz did not affect the delegation of egg laying. The invasion rate was also tracked relative to the control group, which allowed us to conclude that a two-time treatment with amitraz reduced the frequency of mites and a four-time treatment checked the effectiveness. Tailoring the control of *V. destructor* in bee colonies may be an effective measure in the fight against this parasite.

## 1. Introduction

Personalised medicine, a disease treatment method, has gained popularity in the modern world [1]. Recently, personalised medicine has also attracted the interest of veterinary practitioners, and it is successfully used to treat canine neoplasia [2]. However, the applicability of personalised medicine has never been studied in honey bees. *Varroa destructor* mites pose the greatest threat to honey bee colonies [3]. These parasites harm colony health and carry multiple bee viruses [4,5,6,7,8,9,10,11], compromising a colony’s survival. Due to their impact on honey bees, *V. destructor* can be used as a colony collapse marker, especially during winter losses [12,13]. Attempts to eliminate *V. destructor* by beekeepers and scientists in Europe have been ongoing for forty years. Various methods of combating the mites have been implemented during this period, but the problem still needs to be solved. Linking genetic conditions; monitoring the parasite invasion, its biology, and behavior in the bee colony; and the “personalisation”(tailoring) of control may influence the modification of the treatment against the parasite [14].

Knowledge of a patient’s genome is crucial in personalised medicine. Genetic material can influence the progression of disease and the patient’s response to treatment [15]. Mite *V. jacobsoni* invasions in Asiatic honey bee (*A. ceranae*) colonies do not inflict significant losses [16,17] because these bees have developed specific hygiene behaviours [18]. Different subspecies of the Western honey bee have also developed behavioural adjustments, such as improved hygiene, more frequent swarming, and a shorter brood development phase [19,20], to combat invasions [21,22].

Various substances and protocols have been proposed for treating *V. destructor* invasions. The most widely used compounds include amitraz, coumaphos, tau-fluvalinate, thymol, oxalic, and formic acid [23]. Amitraz is arguably the most controversial treatment, widely used in some countries and banned in others. Boncristiani et al. [24] demonstrated that amitraz does not increase the expression of genes responsible for detoxication in bees. Amitraz is fully decomposed within only 10 days [25] and does not exert toxic effects on honey consumers or bee broods. However, multiple studies have shown that mites increasingly resist the “hard” substances used for invasion control [26,27,28]. Amitraz can also compromise bees’ immune response to viral infections [29]. Their grooming behaviour may also be hindered by the use of amitraz [30].

In 2020, we conducted a preliminary study on the personalised treatment of varroosis in 24 Western honey bee colonies. The study aimed to examine the effects of treatment on colonies with a uniform genetic background that live under the same conditions. A personalised treatment was implemented by adjusting amitraz doses to the number of female mites in brood cells and bee queens’ reproductive potential. Before and after treatment, the queens were kept in isolators, and brood combs were removed to control varroosis.

## 2. Materials and Methods

### 2.1. Honey Bee Colonies

Bee colonies were kept in a 120-colony apiary in northern Poland between June and September 2020. In the apiary, Bayvarol (Bayer) was used to control the most recent *V. destructor* invasion in 2019. Twenty-four Western honey bee (*A. mellifera carnica*) packages (each weighing 1.5 kg), certified as Varroa-free, were purchased in June 2020. Each package was placed inside a Dadant bee hive with five frames with a wax foundation. Then, *A. mellifera carnica* Sklenar queens were added to every colony. The queens were previously inseminated with the semen of akin drones. The bee colonies were fed twice with 1 L of 1:2 sucrose–water solution. New frames were added according to need, but the bees were left undisturbed otherwise. The bee colonies were naturally invaded by *V. destructor*.

### 2.2. Treatment

On 2 September, the hives were inspected, and uncapped brood frames were removed. The queens were kept in isolators/clips for 12 days until the workers from the capped brood emerged. Subsequently, the queens were moved to a single frame. Chmara’s isolator was used in scientific research.

After 12 days, the frames containing the broods from each hive were removed again. The area occupied by the capped brood was measured (dm^2^), the caps were removed, and mites were counted under a magnifier with a light source. We created 2 groups of 12 colonies each based on mite counts: group I with less than 20 female mites per brood cell on average, and group II with more than 20 female mites per brood cell on average. Varroosis was treated with one Apiwarol (Biowet Puławy, Poland) fumigation tablet (12.5 mg amitraz) per bee colony, applied past 6 p.m. for 30 min. The groups were divided into subgroups of four colonies each, and each subgroup received a different treatment. Subgroups Ia and IIA received Apiwarol twice within a four-day interval. Subgroups Ib and IIB received Apiwarol four times in two-day intervals. Ic and IIC were the control subgroups that did not receive any treatment. In all subgroups, the day after the last treatment, the queens were again moved to a single frame. After 12 days, brood frames were removed, brood area was measured, and mites were counted. The brood came from a 1-frame isolator.

Screened open-bottom boards were placed inside every hive, and the colonies were treated with one dose of Apiwarol. The boards were removed on the following day, and mites were counted. A detailed treatment protocol is presented in Table 1.

The queens were kept in clips and in isolators to assess their brooding potential. It should be noted that brood removal is also a method of varroosis control.

### 2.3. Mathematical and Statistical Analyses

The calculations were performed in Microsoft Excel. Infestation levels were determined by dividing mite counts by brood area (dm^2^) in each hive. Average values and standard deviation were calculated. Significant differences in brood area and mite counts between subgroups were evaluated between and after treatments by multiple comparison testing in ANOVA. Levene’s test was used to assess the equality of variances. Pairwise comparisons were performed using Student’s *t*-test, and *p* values below 0.05 were regarded as statistically significant.

## 3. Results

Brood area, mite counts, and infestation levels in group I are shown in Table 2. In group I, the brood area was 12.5–18 dm^2^ before treatment and 16–20 dm^2^ after treatment. In all subgroups, the average brood area significantly increased on the second brood removal date: from 14.75 (±1.6) to 18.25 dm2 (±1.35) in subgroup Ia, from 15.13 (±2.07) to 18.75 dm^2^ (±1.03) in subgroup Ib and from 14.75 (±0.75) to 18.38 dm^2^ (±1.08) in subgroup Ic. After treatment, average mite counts significantly increased from 14.5 (±4.27) to 26.75 (±3.83) in subgroup Ia and from 16.75 (±1.48) to 40.5 (±6.42) in subgroup Ic, but decreased from 15.25 (±3.83) to 13.75 (± 2.68) in subgroup Ib (Table 1). Consequently, infestation levels increased from 1.00 (±0.37) to 1.47 (±0.32) in subgroup Ia and from 1.14 (±0.17) to 2.20 (±0.39) in subgroup Ic and decreased from 0.99 (±0.16) to 0.74 (±0.19) in subgroup Ib.

Brood area, mite counts, and infestation levels in group II are shown in Table 3. The brood area was 12.5–18 dm^2^ before treatment and 16–19.5 dm^2^ after treatment. Similar to group I, a significant increase in brood area was observed in group II on the second brood removal date, from 16.13 (±1.42) to 17.5 dm^2^ (±1.12) in subgroup IIA, from 14.25 (±1.25) to 17.5 dm^2^ (±1.12) in subgroup IIB, and from 16.13 (±1.67) to 18.25 dm^2^ (±1.03) in subgroup IIC. No significant differences in brood area were noted between subgroups. After treatment, average mite counts significantly increased from 31.5 (±3.2) to 40.25 (±10.87) in subgroup IIA and from 35.5 (±8.73) to 42.75 (±8.84) in subgroup IIC but decreased from 36 (±8.09) to 22.75 (±3.03) in subgroup IIB. Average infestation levels increased from 1.97 (±0.29) to 2.27 (±0.57) in subgroup IIA and from 2.21 (±0.63) to 2.35 (±0.61) in subgroup IIC, but decreased from 2.56 (±0.77) to 1.3 (±0.26) in subgroup IIB.

The average brood area was somewhat smaller in group I (14.88 dm^2^ ± 1.65) than in group II (15.5 dm^2^ ± 1.78) before treatment, but it was higher in group I (18.45 dm^2^ ± 1.23) than in group II after treatment (17.75 dm^2^ ± 1.2). The average mite counts were higher in group II (34.33 ± 2.47) than in group I (15.5 ± 1.15) before treatment and remained higher after treatment (35.25 ± 10.9 and 27 ± 13.38 in groups II and I, respectively). Infestation levels were significantly higher in subgroups Ia (Δ = 0.47 ± 0.46) and Ic (Δ = 1.06 ± 0.54) than in subgroups IIA (Δ = 0.3 ± 0.72) and IIC (Δ = 0.13 ± 0.61). In contrast, infestation levels were lower in subgroup Ib (Δ = −0.25 ± 0.34) than in subgroup IIB (Δ = −1.25 ± 0.26).

The number of *V. destructor* females on the screened bottom boards is presented in Table 1 and Table 2. The mites found on the screened bottom boards accounted for 66%, 56%, and 68% of total mite counts in subgroups Ia, Ib, and Ic, respectively, and for 59%, 59%, and 58% of total mite counts in subgroups IIA, IIB, and IIC, respectively. Mite counts were highest in subgroups Ic (91.75 ± 18.02) and IIC (68.5 ± 31.63) and lowest in subgroups Ib (68.5 ± 31.63) and IIB (40.75 ± 22.49). The differences were significant between subgroups Ib and Ic but not between subgroups Ia and Ic or subgroups Ia and Ib. Similar results were observed in group II, where mite counts significantly differed between subgroups IIB and IIC but not between subgroups IIA and IIC.

The linear regression plots of mite counts and brood area (fixed factor) are shown in Figure 1, Figure 2, Figure 3, Figure 4, Figure 5, Figure 6, Figure 7 and Figure 8.

## 4. Discussion

The honey bee genome was first published by the Honeybee Genome Sequencing Consortium [31] and has been modified since [32,33]. Unlike relatives such as bumblebees, honey bees have a very high recombination potential, and genomic differences are observed within colonies [34]. Genetic diversity increases honey bees’ resistance against pathogens and slows pathogen spread [35]. The use of queens from local breeders is connected to sustainable productivity and decreased colony losses [36]. The genome of honey bees analysed in the present study was unknown, and it could only be inferred from the common origin of mothers–sisters and drones–brothers. This study investigated the effects of different treatment protocols on closely related colonies living under the same conditions, characterised by similar breeding potential and similar *V. destructor* infestation levels. After treatment, no significant differences in breeding potential were found between subgroups of group I (Ia vs. Ib, Ia vs. Ic, and Ib vs. Ic) or group II (IIA vs. IIB, IIA vs. IIC, and IIB vs. IIC). Different types of treatment did not induce significant differences in the breeding potential of the corresponding subgroups of groups I and II either. These results suggest that Apiwarol did not negatively or positively affect honey bee reproduction.

The queens were isolated on a single frame to assess breeding potential, which was assumed to be similar in all colonies (no significant differences). Before treatment, the mean brood area was greater in colonies infested by more than 20 female mites (group II), which could be expected because a higher number of brood cells is likely to attract a higher number of female mites. After treatment, the mean brood area was larger in group I than in group II, but the observed differences were insignificant. However, the statistical analysis results could be flawed due to the small size of the subgroups. The impact of amitraz on queens’ breeding potential has not been examined to date. *Varroa destructor* itself does not appear to influence breeding in honey bee colonies. According to Rusert et al. [37], these parasites do not compromise honey bees’ mating success either.

According to the manufacturer’s instructions, Apiwarol should be administered two or three times over four to six days. After treatment, mite counts and infestation levels increased in subgroups Ia and IIA, where Apiwarol was applied twice. After four treatments (subgroups Ib and IIB), infestation levels significantly decreased relative to those of the control group. These results suggest that Apiwarol should be administered more than twice to obtain a satisfactory outcome, even in less-infested colonies. However, when applied four times in two-day intervals, the treatment effectively reduced infestation levels.

Screened bottom boards were used in the study to estimate the number of mites remaining on the workers. After treatment, the mite counts on boards were positively corelated with the mite counts in brood cells. Regardless of the applied treatment, mite counts were higher on the boards than in brood cells in all subgroups. The proportion of mites found on screened boards in total mite counts did not significantly differ between control subgroups (Ic, IIC) and the subgroups that received treatment (subgroups Ia, Ib, IIA, and IIB were fumigated once with Apiwarol at the end of the experiment).

**Figure 1:**
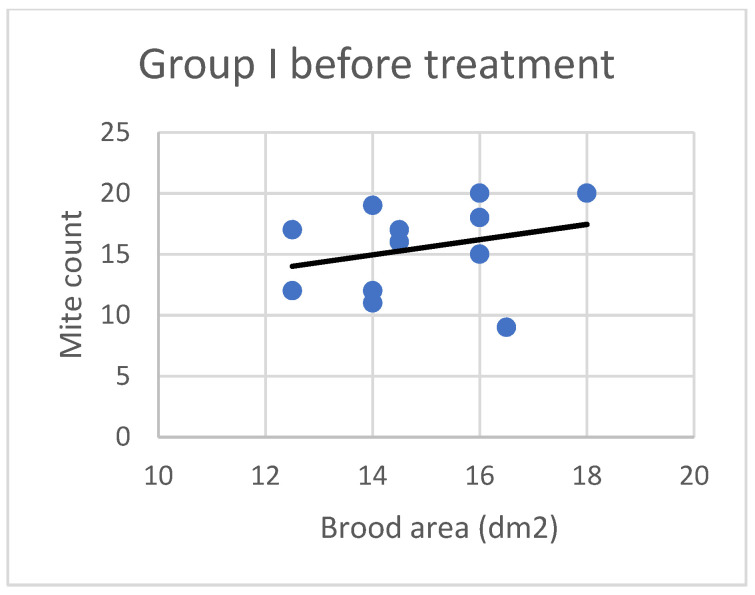
Linear regression plots of brood area (fixed factor) and mite counts in Group I before treatment.

**Figure 2:**
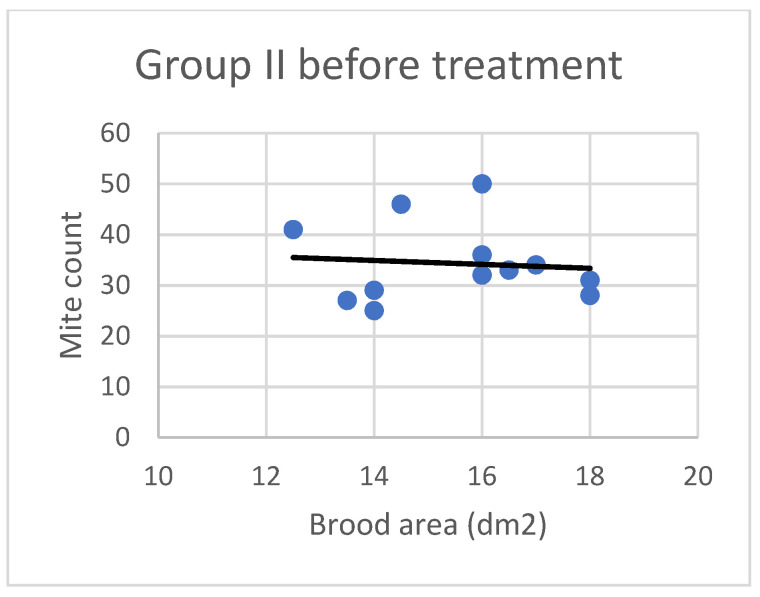
Linear regression plots of brood area (fixed factor) and mite counts in Group II before treatment.

**Figure 3:**
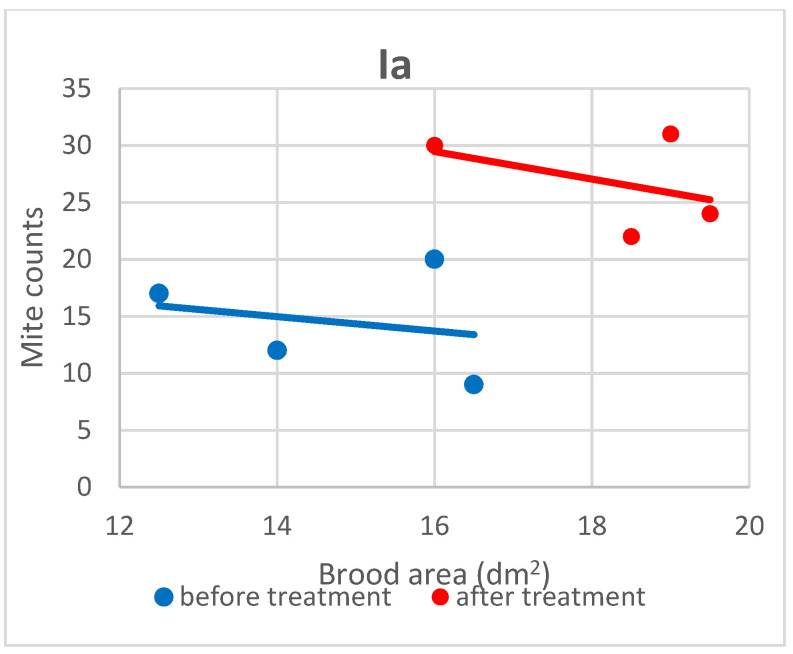
Linear regression plots of brood area (fixed factor) and mite counts in subgroups Ia before and after treatment.

**Figure 4:**
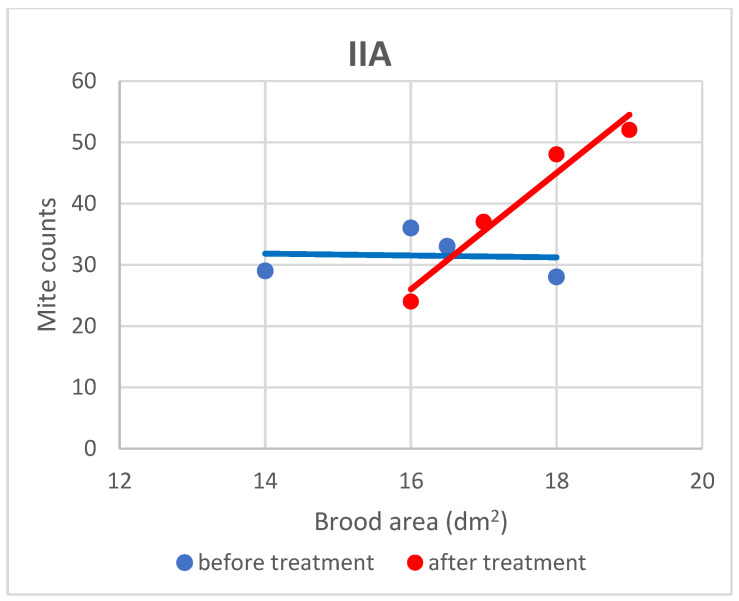
Linear regression plots of brood area (fixed factor) and mite counts in subgroup IIA before and after treatment.

**Figure 5:**
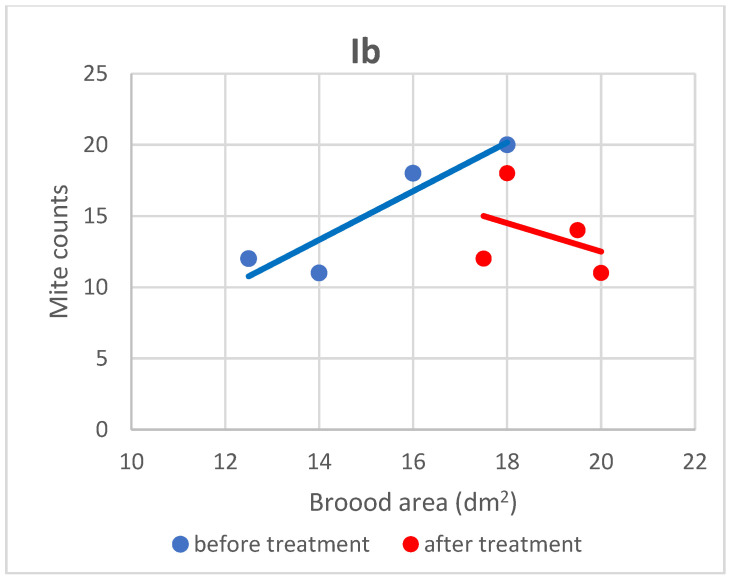
Linear regression plots of brood area (fixed factor) and mite counts in subgroup Ib before and after treatment.

**Figure 6:**
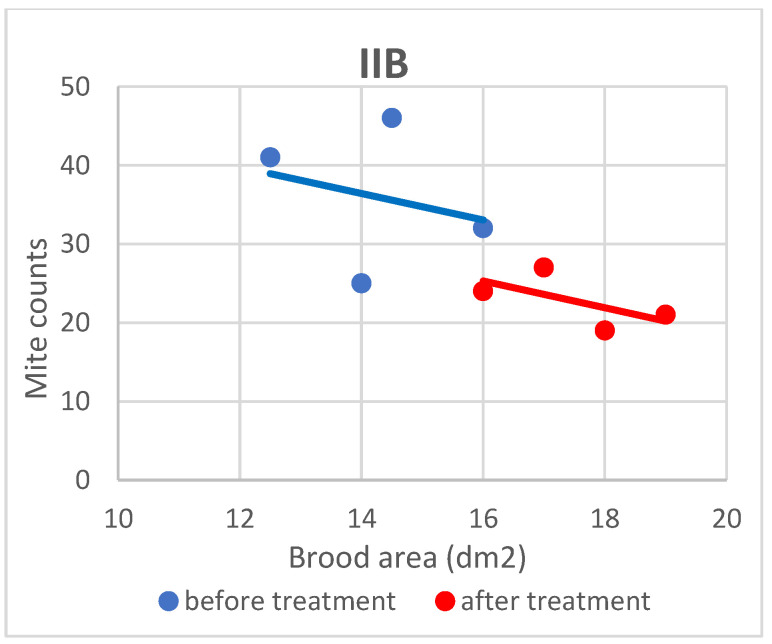
Linear regression plots of brood area (fixed factor) and mite counts in subgroup IIB before and after treatment.

**Figure 7:**
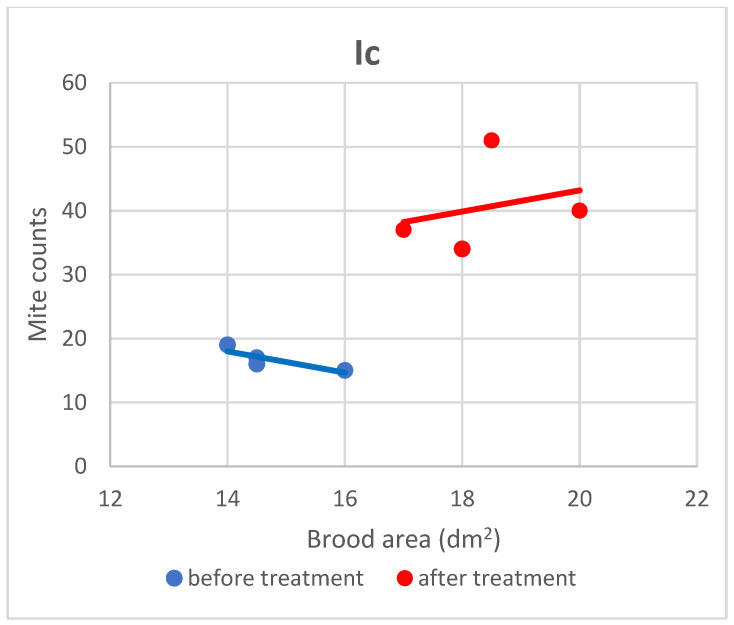
Linear regression plots of brood area (fixed factor) and mite counts in subgroup Ic before and after treatment.

**Figure 8:**
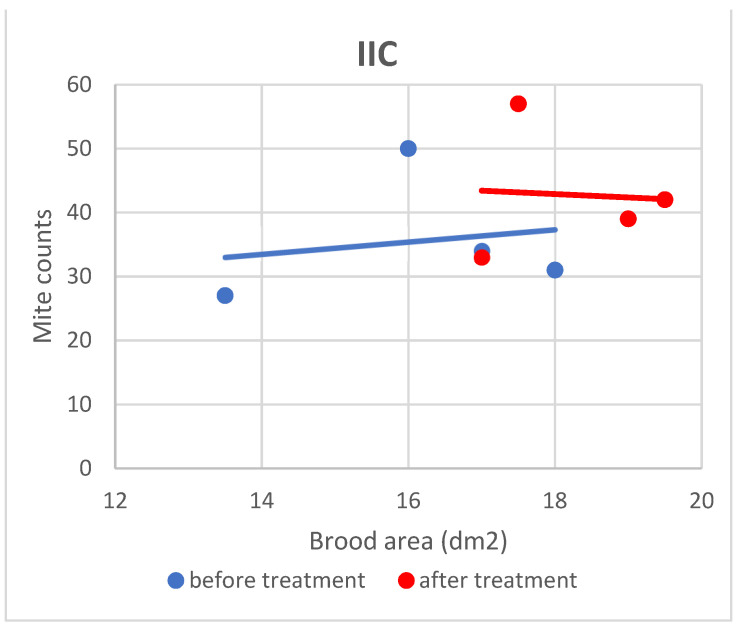
Linear regression plots of brood area (fixed factor) and mite counts in subgroup IIC before and after treatment.

Based on the present findings, the correlation between brood area and infestation levels could not be clearly established (Figure 3, Figure 4, Figure 5, Figure 6, Figure 7 and Figure 8). The results noted in groups I and II before treatment (Figure 1 and Figure 2) indicate that greater brood area was associated with higher mite counts in colonies infested with less than 21 female mites. In contrast, in group II, brood area was smaller in colonies with higher mite counts. The linear regression analysis was not performed separately for each subgroup due to the small number of colonies.

In addition to the genome, the queens’ breeding behaviour can also be influenced by age because younger queens lay more eggs [38]. In a study by Gregorc and Planinc [39], colonies were fumigated once with three drops of 12.5% amitraz solution. The average mite count on screened bottom boards was determined to be 50.61(±36.11) per colony, similar to the results noted in the present study. Similar mite counts were observed after two thymol treatments (Apiguard and Thymovar). Sammataro et al. [27], Mathieu and Faucon [40], and Sajid et al. [41] assessed the efficacy of fluvalinate, flumethrin, amitraz, formic acid, and oxalic acid in reducing mite infestation levels in adult honey bees.

## 5. Conclusions

The removed brood was a reliable source of knowledge about *V. destructor* infestation, and brood removal proved to be an effective treatment protocol. Recent studies have shown that drone brood removal can also increase honey production because more workers are available for foraging. Further research into the genome of the honey bee is needed to develop an effective method of varroosis control. The results of the present study suggest that mite counts should be determined before treatment because a drug’s efficacy depends on infestation levels. In addition, Varroa monitoring should be emphasised. The consequence of personalised control of *V. destructor* in bee colonies, especially when using preparations in the form of smoke, spray, or hanging strips several times during the beekeeping season, is likely a reduction in the number of control treatments. It is also essential to avoid the reported drug resistance of this mite to many substances and to eliminate the residues of these substances in bee products.

## Figures and Tables

**Table 1:** Treatment protocol.

June		
24 packs, *V. destructor* (−) beehive with a Dadant frame with a wax foundation (5 frames) young mothers (sisters) without interfering with development		
**September**		
elimination of open brood queen in a cage (release of *V. destructor* from capped brood) insertion of an isolator (wax foundation + mother) (cumulation of *V. destructor* and reproductive force)		
after 12 days	brood surface measurement (isolator) determination of the number of *V. destructor* (isolator)	
formation of groups	I 12 colonies (1–20 *V. destructor*)	II 12 colonies (<21 *V. destructor*)
**Personalised treatment**		
Apiwarol dose: 1 tab./colony/1 treatment/for 30 min. after 18 h		
2 treatments every 4 days	4 colonies (a)	4 colonies (A)
4 treatments every 2 days	4 colonies (b)	4 colonies (B)
without treatment	4 colonies (c)	4 colonies (C)
after the last treatment	open brood was removed queen in a cage (release of *V. destructor* from capped brood) Isolator inserted (wax foundation + mother) cumulative *V. destructor* and reproductive force	
after 12 days	brood surface measurement (isolator) number of *V. destructor* (brood isolator) number of *V. destructor* on workers (bottom inserts + 1× Apiwarol)	

**Table 2:** Brood area (dm^2^) and mite counts in group I.

Subgroup	Colony Number	Brood Area (dm^2^)		Mite Counts		V/dm^2^		ΔV/dm^2^	Mite Counts on Screened Bottom Boards
		Before Treatment	After Treatment	Before Treatment	After Treatment	Before Treatment	After Treatment		
Ia	1	14	18.5	12	22	0.86	1.19	0.33	46
	2	12.5	16	17	30	1.36	1.86	0.50	32
	3	16.5	19	9	31	0.55	1.63	1.08	63
	4	16	19.5	20	24	1.25	1.23	−0.02	80
	Av/SD	14.75 (±1.6)	18.25 (±1.35)	14.5 (±4.27)	26.75 (±3.83)	1.00 (±0.37)	1.47 (±0.32)	0.47 (±0.46)	55.25 (± 18.02)
Ib	5	14	18	11	18	0.79	1	0.21	21
	6	16	17.5	18	12	1.13	0.69	−0.44	8
	7	18	20	20	11	1.11	0.55	−0.56	34
	8	12.5	19.5	12	14	0.96	0.72	−0.24	17
	Av/SD	15.13 (±2.07)	18.75 (±1.03)	15.25 (±3.83)	13.75 (±2.68)	0.99 (±0.16)	0.74 (±0.19)	−0.25 (±0.34)	20 (± 9.35)
Ic	9	14.5	17	16	37	1.1	2.18	1.08	73
	10	14	20	19	40	1.36	2	0.64	104
	11	14.5	18	17	34	1.18	1.89	0.71	57
	12	16	18.5	15	51	0.94	2.76	1.82	133
	Av/SD	14.75 (±0.75)	18.38 (±1.08)	16.75 (±1.48)	40.5 (±6.42)	1.14 (±0.17)	2.20 (±0.39)	1.06 (±0.54)	91.75 (± 29.2)

**Table 3:** Brood area (dm^2^) and mite counts in group II.

Subgroup	Colony Number	Brood Area (dm^2^)		Mite Counts		V/dm^2^		ΔV/dm^2^	Mite Counts on Screened Bottom Boards
		Before Treatment	After Treatment	Before Treatment	After Treatment	Before Treatment	After Treatment		
IIA	1	16.5	17	33	37	2	2.18	0.18	56
	2	14	16	29	24	2.07	1.5	−0.57	71
	3	16	18	36	48	2.25	2.67	0.42	27
	4	18	19	28	52	1.56	2.74	1.18	92
	Av/SD	16.13 (±1.42)	17.5 (±1.12)	31.5 (±3.2)	40.25 (±10.87)	1.97 (±0.29)	2.27 (±0.57)	0.30 (±0.72)	61.5 (± 23.67)
IIB	5	12.5	16	41	24	3.28	1.5	−1.78	36
	6	16	17	32	27	2	1.56	−0.44	75
	7	14	18	25	19	1.79	1.06	−0.73	40
	8	14.5	19	46	21	3.17	1.1	−2.07	12
	Av/SD	14.25 (±1.25)	17.5 (±1.12)	36 (±8.09)	22.75 (±3.03)	2.56 (±0.77)	1.3 (±0.26)	−1.25 (±0.26)	40.75 (± 22.49)
IIC	9	16	17.5	50	57	3.13	3.26	0.13	84
	10	18	19	31	39	1.72	2.05	0.33	31
	11	13.5	17	27	33	2	1.94	−0.06	112
	12	17	19.5	34	42	2	2.15	0.15	47
	Av/SD	16.13 (±1.67)	18.25 (±1.03)	35.5 (±8.73)	42.75 (±8.84)	2.213 (±0.63)	2.35 (±0.61)	0.13 (±0.61)	68.5 (± 31.63)

## Data Availability

Detailed results can be obtained upon request from the first author.
